# Comparative biomechanical analysis of equine accessory carpal bone fracture repair: Cortical screws in lag fashion versus X‐plate technique

**DOI:** 10.1111/vsu.70071

**Published:** 2025-12-21

**Authors:** Jennifer Gernhardt, Thomas Reuter, Kathrin Mählmann, Nicole Schulze, Christoph J. Lischer

**Affiliations:** ^1^ Equine Clinic Freie Universität Berlin Berlin Germany; ^2^ ICM‐Institut Chemnitzer Maschinen‐ und Anlagenbau e.V Chemnitz Germany

## Abstract

**Objective:**

To compare the feasibility and biomechanical stability of two surgical techniques for fixation of vertical plane fractures of the accessory carpal bone (ACB).

**Study design:**

Randomized experimental ex vivo study.

**Sample population:**

Eight equine accessory carpal bones were included in a control group. A total of 20 equine cadaveric forelimbs were randomly assigned into two groups (*n* = 10 per group).

**Methods:**

Vertical plane fractures were created palmar to the extensor sulcus using an oscillating saw. In Group 1 (CS), fractures were stabilized with two 4.5 mm cortical screws in lag fashion. In Group 2 (XP), fixation included one 4.5 mm cortical screw in lag fashion and a laterally applied angular stable X‐plate with four 2.7 mm locking screws. Control ACBs were excised and tested under axial compression using a four‐column testing machine. Postoperative specimens in Groups CS and XP were tested under the same conditions. The failure mode was assessed radiographically.

**Results:**

The mean maximum strength of native bone was 11.26 (±2.14) kN. Two constructs per group were excluded due to cortical screw protrusion. No difference in failure load was observed (CS: 6.82 [±2.34] kN; XP: 8.02 [±1.10] kN; *p* = .7558). Failure mode analysis revealed a greater fracture gap size (*p* = .0039) and implant bending in CS specimens (*p* = 1.074e‐7).

**Conclusion:**

Both techniques were feasible, though neither restored native bone strength.

**Clinical significance:**

A lateral X‐plate with a single cortical screw demonstrated equivalent biomechanical performance to two cortical screws and was technically less demanding, offering a simpler fixation option for ACB fractures.

## 1 INTRODUCTION

Fractures of the accessory carpal bone (ACB) are mostly seen in racehorses but may also occur in other types of horses and under various circumstances,^1^ such as falls, particularly during racing over fences.^1^, ^2^, ^3^, ^4^ The bone typically fractures in a vertical plane, often with varying degrees of comminution,^5^ whereas horizontal fractures are rare.^6^, ^7^ Several theories have been proposed regarding the development of these fractures; however, the etiopathogenesis remains incompletely understood.^1^

The treatment of ACB fractures remains a subject of debate in the literature.^5^, ^8^

Most textbooks currently recommend conservative treatment and endoscopic removal of small fragments via the carpal sheath or palmar carpal arthroscopy.^9^ Although conservative treatment is generally associated with a good prognosis for return to soundness, it frequently results in bony non‐union.^8^, ^10^ To date, no published data are available directly comparing conservative with surgical treatment.

**FIGURE 1:**
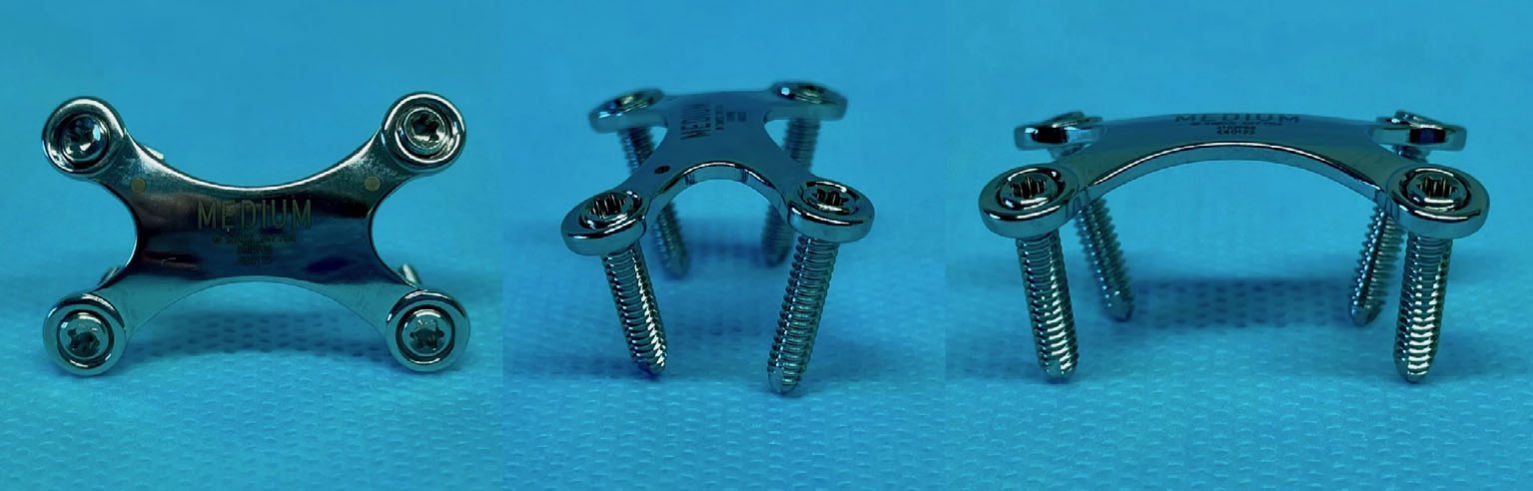
Medium angular stable X‐plate (DePuy Synthes, Johnson & Johnson MedTech) with four 2.7 mm locking head screws. The plate's convex contour matches the lateral side of the accessory carpal bone (ACB).

The high incidence of fractures occurring during exercise suggests a relationship with biomechanical forces transmitted through the ligamentous attachments.^11^ Constant traction from tendons and ligaments leads to movement at the fracture site and may impair healing. Internal fixation with interfragmentary compression improves bone healing.^12^, ^13^ Fracture repair using cortical screws in lag fashion provides optimal interfragmentary compression. However, a single screw does not offer sufficient rotational stability,^14^ and the placement of two or more screws is technically difficult, particularly in smaller horses. There is a substantial risk of screw penetration into the carpal canal medially or lateral cortical breach, both of which may compromise the stability of the fracture fixation.^5^ Recently, surgical repair has been described in case reports: one involving computer‐assisted placement of cortical screws in lag fashion,^15^ and another combining a talonavicular fusion plate with screws in lag fashion.^16^ Nevertheless, vertical plane fractures of the ACB are rarely surgically repaired because of the difficulty of placing screws in lag fashion in a convex‐shaped bone, and data on the biomechanical stability of ACB fracture repair remain lacking.^8^, ^17^ The X‐plate, which is currently used in human foot surgery,^18^ offers a potential alternative. Its convex contour and configuration of four 2.7 mm locking head screws conform closely to the convex lateral surface of the ACB (Figure 1). This anatomical fit is expected to provide sufficient construct stability while neutralizing tensile forces acting on the lateral tension side of the bone.^19^ To the authors' knowledge, biomechanical testing comparing two different surgical techniques for repairing vertical plane fractures of the ACB has not been previously reported.

The aim of this study was to compare the feasibility and biomechanical stability of two surgical techniques for fixation of simple vertical plane fractures of the accessory carpal bone in an axial compression model.

The authors hypothesized that a construct combining a laterally applied plate with a single cortical screw in lag fashion would provide mechanical stability equivalent to, or greater than, fixation with two cortical screws in lag fashion.

## 2 MATERIALS AND METHODS

### 2.1 Collection of specimens

A total of 16 horses were euthanized for reasons unrelated to carpal joint pathology. Written owner consent was obtained for postmortem use, and the study was approved by the local Committee on Research Ethics of the State Office for Health and Social Affairs (approval no.: StN 015/23). A control group of eight ACBs was collected from these animals, all of which were all Warmblood horses, including three geldings and one mare with a mean age of 14 (±1.53) years and a mean bodyweight of 614.66 (±135.74) kg. Age and bodyweight data were unavailable for one horse. The bones were dissected free of soft tissues for biomechanical testing. Although removal of soft tissue alters the native biomechanical environment of the ACB, it was necessary to standardize the experimental setup.

The remaining 20 equine cadaveric forelimbs (9 right, 11 left) were dissected at the cubital joint and stored in an extended position. The limbs were obtained from 12 horses (8 geldings and 4 mares) with a mean age of 17 (±5.84) years. The sample included nine Warmbloods, one Thoroughbred, one American Quarter Horse, and one pony. Mean bodyweight was 569.22 (±82.56) kg. The bodyweight of three horses was unknown. Specimens were frozen at −20°C and thawed at room temperature for 24 h prior to fracture creation.

### 2.2 Fracture fixation

**FIGURE 2:**
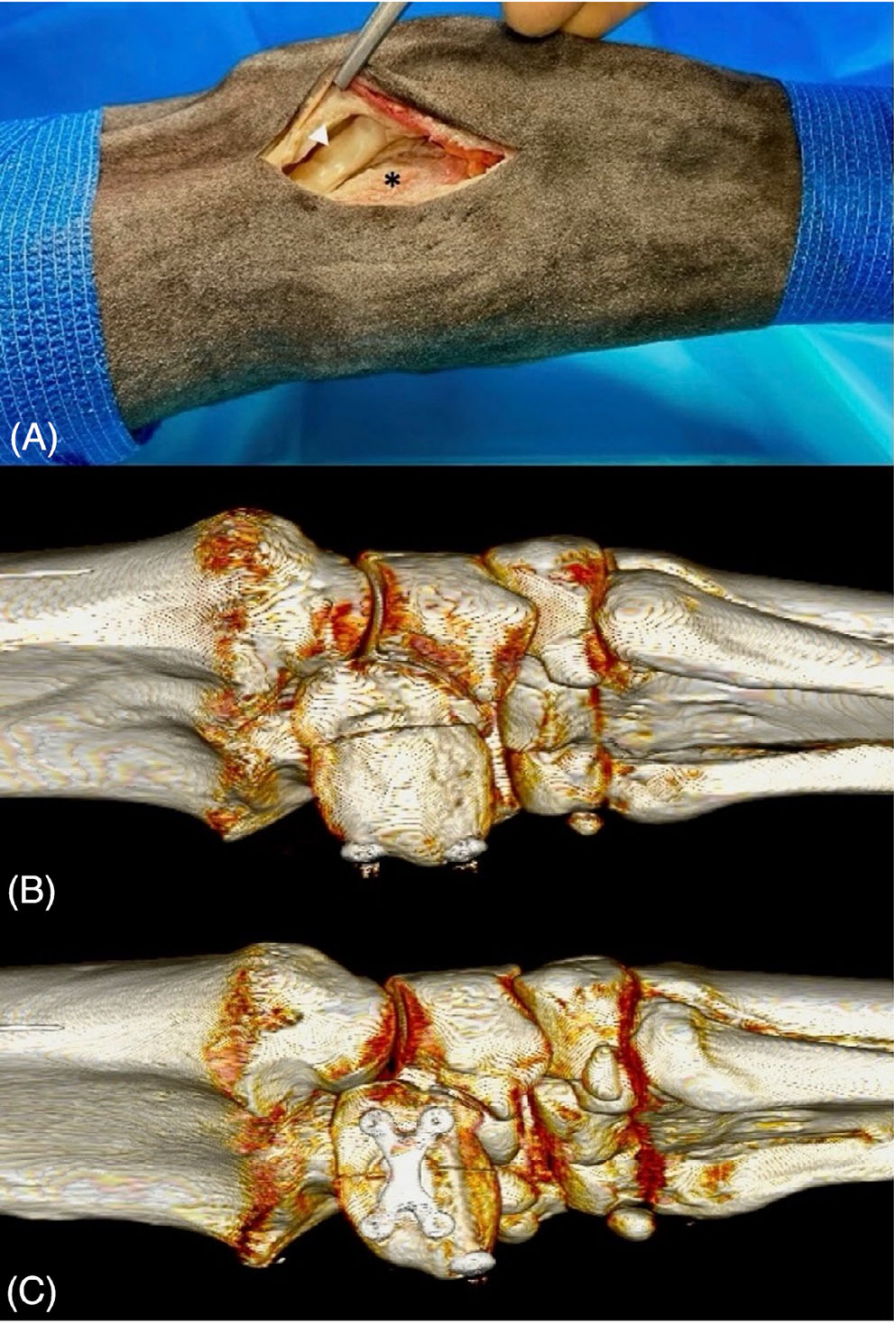
Surgical approach to the lateral surface of the accessory carpal bone (ACB), with proximal oriented to the left. The tendon of the extensor carpi ulnaris muscle is indicated by the white arrowhead; the ACB is marked with an asterisk. Postoperative three‐dimensional volume rendering in the same orientation of a specimen repaired using two cortical screws in lag fashion inserted via stab incisions at the palmar margin of the ACB. Postoperative three‐dimensional volume rendering in the same orientation of a specimen repaired using a single cortical screw in lag fashion and medium X‐plate laterally, which was fixated with four 2.7 mm locking head screws. The surgical approach for the cortical screw was at the palmar border of the ACB. The X‐plate was applied via the surgical approach shown in A, which was extended by sharp dissection of the lateral soft tissues over the ACB, primarily consisting of fascia attached to the bone, to enable direct contact between the plate and the lateral cortical surface.

The limbs were positioned in full extension with the lateral side facing upward. The hoof was clamped in a screw fixture, and the radius was secured to the table with adhesive tape. Fracture fixation was performed by the same board‐certified surgeon (CL). The ACB was approached via an approximately 5 cm long vertical skin incision over the extensor carpi ulnaris muscle (Figure 2). The tendon sheath of the extensor carpi ulnaris muscle was incised to allow mobilization of the tendon. To simulate vertical plane fractures, a standardized cut was made palmar to the extensor sulcus using an oscillating saw. Complete separation of the bone was confirmed by inserting a periosteal elevator into the fracture gap and mobilizing the palmar fragment. The skin incision was not closed prior fracture fixation.

First, the fracture fragments were repositioned using two pointed reduction forceps. A lateromedial radiograph confirmed satisfactory reduction of the fracture line, which marked the starting point for time measurement.

**FIGURE 3:**
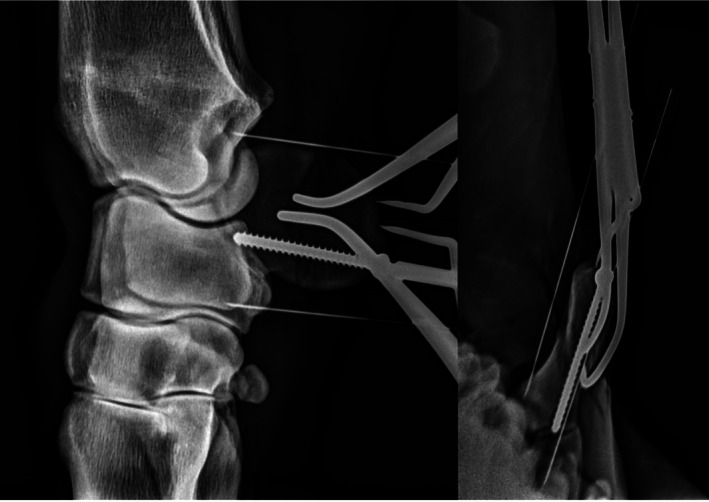
Intraoperative radiographs of repairing a vertical plane fracture of the accessory carpal bone (ACB). Left image: Radiograph in latero‐medial projection. The insertion of a cortical screw in lag fashion from palmar to dorsal into the distal portion of the ACB was guided by spinal needles which were placed proximally and distally to the ACB for drill orientation. Right image: Radiograph in D80°Pr30°L PaDiMO. Two spinal needles, one proximal and one distal to the ACB, were used to orientate the cortical screw, which was inserted between the lateral and medial cortex of the ACB in a palmar‐to‐dorsal direction.

The limbs were divided into two equal groups. The specimens in Group 1 (CS) were surgically repaired using two 4.5 mm cortical screws inserted horizontally in lag fashion, directed from palmar to dorsal. A hypodermic needle (20 gauge × 1 ½″ 0.90 × 40 mm) was inserted palmarly, midway between the proximal and distal borders of the ACB, to mark the horizontal midline of the bone. Two spinal needles (20 gauge × 3 1/2″ 0.90 × 90 mm) were then inserted along the proximal and distal borders of the ACB, perpendicular to the fracture line. These spinal needles were placed in the estimated direction of the screws from palmar to dorsal and were advanced to the palmar radius or ulnar carpal bone. Care was taken to insert the needles slightly lateral to the palmar margin to avoid later penetration of the medial cortex by the cortical screwsdue to the convex shape of the bone. Lateromedial and D80°Pr30°L‐PaDiMO views were used to confirm needle orientation.^20^ Optimal alignment on lateromedial radiographs was defined by centrally placed spinal needles in the proximal and distal halves of the bone, orientated perpendicular to the fracture line. In D80°Pr30°L‐PaDiMO views, correct placement was confirmed when the needles were projected between the medial and lateral cortices of the ACB (Figure 3).

Two 4.5 mm cortical screws (DePuy Synthes, Johnson & Johnson MedTech) in lag fashion were inserted using standard technique.

**FIGURE 4:**
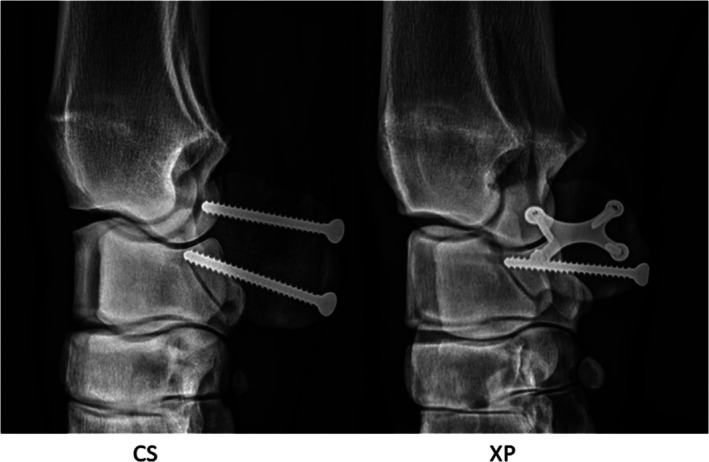
Postoperative radiographs of the accessory carpal bone (ACB) following fracture fixation. Left: Two 4.5 mm cortical screws in lag fashion (CS). Right: Medium X‐plate and 4.5 mm cortical screw in lag fashion (XP).

Fracture fixation in Group 2 (XP) consisted of a 4.5 mm cortical screw placed in lag fashion and a medium angular stable X‐plate fixed laterally to the ACBusing four 2.7 mm self‐tapping locking head screws (DePuy Synthes, Johnson & Johnson MedTech, Norderstedt, Germany) (Figure 4). The existing skin incision was extended by performing sharp dissection of the lateral soft tissues over the ACB, primarily consisting of fascia attached to the bone, to enable direct contact between the plate and the lateral cortical surface. The cortical screw was inserted into the distal third of the ACB, as previously described.

Following fracture reduction by tightening of the cortical screw, the X‐plate was positioned in the proximal two‐thirds of the ACB, with its center aligned over the fracture line.

Correct positioning was confirmed radiographically in lateromedial view. The X‐plate was secured by first drilling the palmar‐distal hole using a 2.0 mm drill bit until the medial cortex was penetrated. A 2.7 mm self‐tapping locking head screw of appropriate length was inserted. Plate position was verified with a lateromedial radiograph. The remaining three holes were drilled, and the screws inserted in the same manner. In both groups, the final radiograph marked the end point of time measurement. All limbs were subsequently examined by computed tomography (Canon Medical Systems, 32‐slice detector, Amstelveen, Netherlands) with a field‐of‐view of 512 × 512 pixels, 135 kV, 370 mA and 1 mm slice thickness.

### 2.3 Biomechanical testing

Subsequently, all ACBs were excised, and all soft tissues were removed. Inclusion criteria for biomechanical testing were adequate fracture reduction and compression, with no screw penetration of the medial or lateral cortex.

**FIGURE 5:**
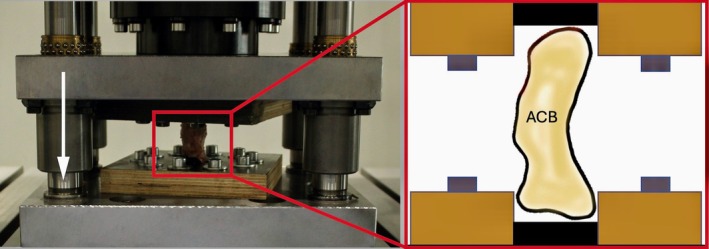
Photograph of the four‐column testing machine used for axial compression testing (left). The schematic illustration (right) shows the accessory carpal bone (ACB) secured palmarly (top) and dorsally (bottom) to enable standardized loading in a palmarodorsal direction. The white arrow indicates the direction of load.

Specimens were stored at −20°C and thawed for 12 h prior to biomechanical testing. The control group was tested first. Following, the specimens of group CS and XP were tested in the same way. A customized construction four‐column testing machine (PräFüMa, Hegewald & PeschkeMeß‐ und Prüftechnik GmbH, Nossen, Germany) with a 100 kN force sensor (resolution 2 N, measurement uncertainty 50 N) was used to determine the maximum strength (force‐to‐failure) of the constructs by quasi‐static uniaxial compression tests in a palmarodorsal direction (Figure 5). To stabilize the specimen and to avoid direct loading on the implants, washers were placed palmarly and dorsally. To prevent the bones from slipping in the fixture, specimens were preconditioned by loading five times at a compression rate of 100 N/s up to 2 kN. Compression testing was then performed at a constant speed of 1 mm/s.

### 2.4 Statistical analysis

Descriptive statistical analysis was reported for cortical screw length, operative time, failure mode, fracture gap size, stiffness, and maximum strength. The Grubbs test (*p* < .05) was used to identify outliers in the data. All measurement data were independent. Normality was assessed using the Shapiro–Wilk test (*p* > .05), and homogeneity of variance was tested using Levene's test (*p* > .05). Failure mode values did not follow a normal distribution according to the Shapiro–Wilk test (CS *p* = 1.385e‐8; XP *p* = 4.604e‐5), and an inhomogeneity of variance according to the Levene's test (*p* = .0075), so a Mann–Whitney U test was applied. Stiffness values failed to meet the assumption of homogeneity of variance (*p* = .0067); therefore, a Kruskal–Wallis ANOVA was performed, followed by Dunn's post hoc test with Bonferroni‐adjusted *p*‐values to compare stiffness between groups.^21^

All other variables showed normal distribution and homogeneity of variance and could therefore be analyzed using a *t*‐test and one‐way ANOVA. A one‐way ANOVA, followed by an unpaired *t*‐test with Bonferroni‐adjusted *p*‐values, was performed to assess differences in maximum strength between groups. An analysis of covariance (ANCOVA) was conducted to evaluate the influence of horse bodyweight on maximum strength. The significance level of all tests was *p* < .05. In addition, the Cohen effect size was calculated for the corresponding significance tests and interpreted according to the effect limits.

Failure mode assessment on post‐test radiographs was independently performed by four veterinarians: two board‐certified surgeons, one experienced surgeon, and one surgery resident. The initial evaluation included identification of implant pull‐out, breakage, or bending, as well as assessment of bone damage, fracture gap size, and fragment displacement. Since no implant pull‐out or breakage was observed in any specimen, the authors adopted a failure grading system based on implant deformation and fragment dislocation.

The final scoring system comprised three grades: Grade 1 was defined as mild bending of the implants. Grade 2 was characterized by moderate bending of the implants and mild dislocation of the palmar fragment. Grade 3 was defined by severe bending of the implants accompanied by moderate to severe dislocation of the palmar fragment.

Interrater reliability was calculated using Fleiss' kappa to assess the strength of agreement of the classified failure mode (assessment of the bending of the implants). The assessment of agreement between evaluators is based on the systematization according to Landis et al. Theoretically, it can range from perfect (1) to poor (0). The gradations are in increments of 0.2.^22^ Fracture gap size was measured by the surgery resident using a digital caliper gauge with an accuracy of ±0.1 mm (FORUM, E/D/E, Wuppertal, Germany). Measurements were categorized into three groups: <3 mm, 3–6 mm, and >6 mm.

Collected data were recorded in Excel 2025 Version 16.96.1 (Microsoft Inc., Redmond, Washington) and descriptive statistics were generated. Statistical analyses were performed using MATLAB 2025a (The MathWorks Inc., Natick, Massachusetts) and RStudio 2024.12.0 with R Version 4.4.2 (R Foundation for Statistical Computing, Vienna, Austria).^23^, ^24^, ^25^, ^26^, ^27^, ^28^

## 3 RESULTS

Radiographic and CT evaluation confirmed satisfactory fracture reduction of the ACB in all cases. Prior to insertion, the required cortical screw length was measured. The mean screw length was 35.3 (±4.25) mm. The mean surgical time is listed in Table 1. For Group CS was 31 (±16.18) min while that for Group XP was 43.75 (±10.66) min. After exclusion of one outlier (66 min, *p* = .0362) from the CS group, the *t*‐test showed a shorter operation time than in XP (*p* = .0037) with a strong effect size of *d* = 1.842.^29^

**TABLE 1:** Operating times for surgical fixation of vertical plane fractures of the ACB using either two cortical screws in lag fashion (CS) or a combination of a lateral angular stable X‐plate with a single cortical screw in lag fashion (XP).

Group	Operating time (minutes)	Mean (SD)
CS	12	31 (±16.18)
	27	
	32	
	66	
	32	
	27	
XP	35	43.75 (±10.66)
	17	
	53	
	34	
	59	
	37	
	28	
	42	
	44	
	53	

*Note*: Values are presented as individual measurements (minutes) for each specimen, with group mean ± SD.

Prior to biomechanical testing, four of the 20 constructs were excluded for not meeting inclusion criteria. In Group CS, this involved one Warmblood mare and one pony mare. In the Warmblood mare, both screws in the same specimen perforated the medial cortex. In the pony mare, the thread hole partially breached the lateral cortex. In Group XP, both ACBs from the same Thoroughbred mare were excluded due to medial cortical perforation by the distal screw. Consequently, eight specimens per group were available for biomechanical testing.

**FIGURE 6:**
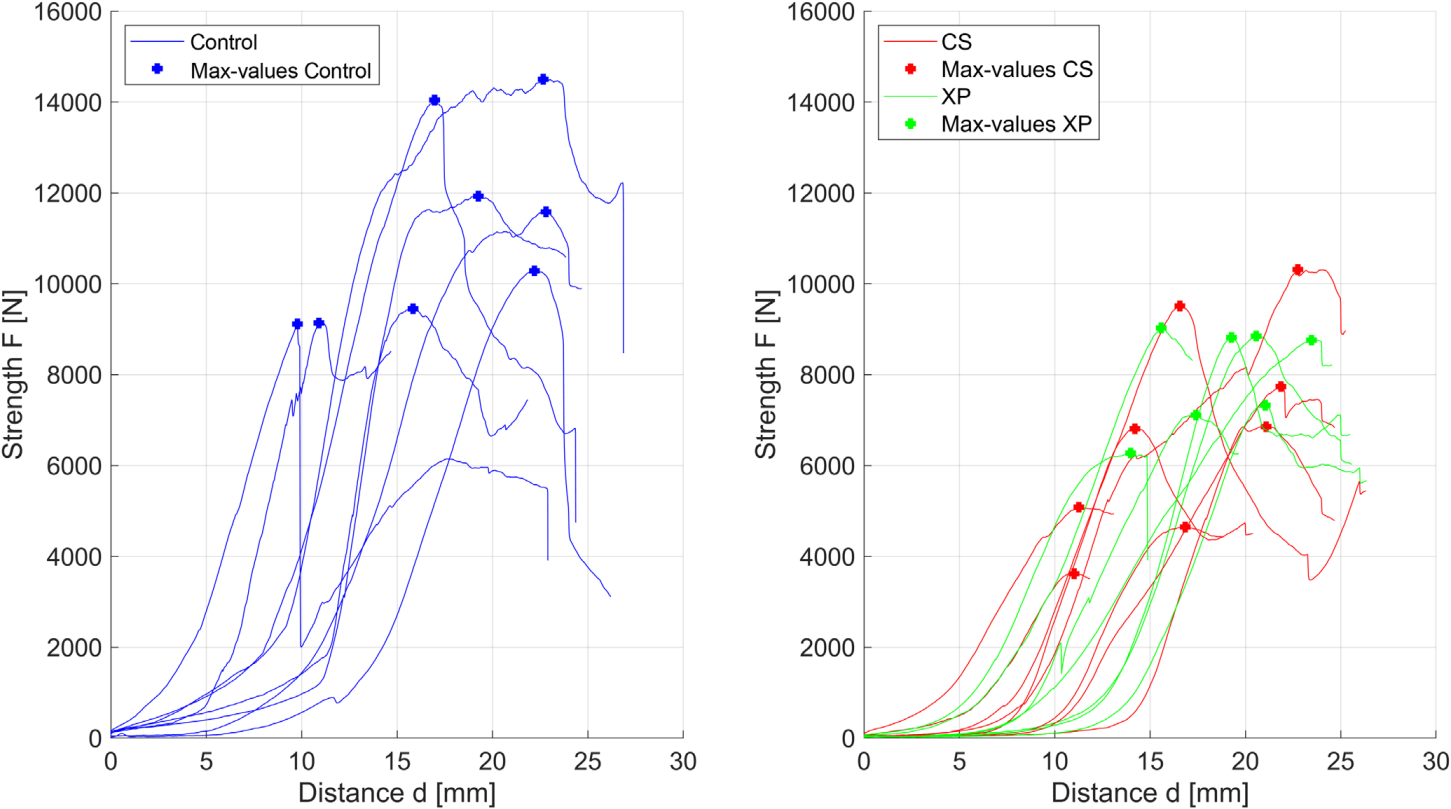
Summary of quasi‐static uniaxial compression tests in the palmarodorsal direction of the accessory carpal bone (ACB) to determine the maximum strength (force to failure). Left: Testing of the native bone (control group). Right: Testing of fractured specimens stabilized with cortical screws in lag fashion (CS, red) and specimens repaired with an X‐plate and a cortical screw in lag fashion (XP, green) (dot‐force to failure, line‐force‐displacement curve).

**FIGURE 7:**
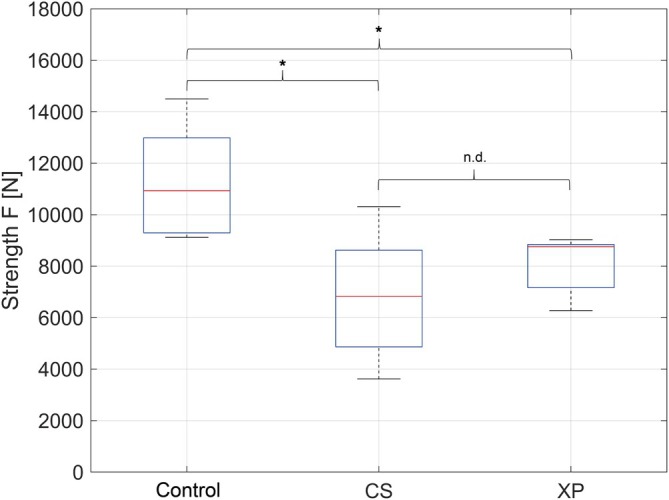
Comparison of the statistical distribution of maximum strength (force to failure) between control group, fracture fixation with cortical screws in lag fashion (CS) and X‐plate and cortical screw in lag fashion (XP). (n.d., no difference *p* > .05; *significant differences *p* < .05; *p*‐value adjustment method: Bonferroni).

Compression testing by quasi‐static uniaxial loading in a palmarodorsal direction demonstrated a mean maximum strength for Group CS of 6.82(±2.34) kN and 8.02 (±1.10) kN for Group XP. The control group (native bone) exhibited a mean maximum strength of 11.26 (±2.14) kN (Figure 6). The maximum strength between CS and XP showed no difference (*p* = .7558). However, the maximum strength of both constructs was less than the native bones (CS *p* = .0007; XP *p* = .0145). These differences indicate a strong effect size of *d* = 1.977 and *d* = 1.889, respectively. The boxplot diagram (Figure 7) indicated that the constructs of XP tended to have higher maximum strength values and less variation in the data compared to CS.

The mean stiffness was 943.2 (±243.8) N/mm for CS and 1040.1 (±253.1) N/mm for XP. The control group demonstrated a mean stiffness of 1884.4 (±587.1) N/mm (Figure S1). Statistical comparison between CS and XP revealed no difference (*p* = 1). The stiffness of both fixation constructs was lower than that of the native bone (CS: *p* = .0018; XP: *p* = .0118). These differences were associated with strong effect sizes (CS: *r* = 0.857; XP: *r* = 0.745) (Figure S2).^29^ A comparison between maximum strength and stiffness showed a positive linear relationship with *r* = 0.67 (Figure S3).

An analysis of covariance (ANCOVA) was conducted to evaluate the effect of group (native vs. CS; native vs. XP) on maximum strength while controlling for horse bodyweight. Assumptions of normality (*p* = .6134), homogeneity of variances (*p* = .2435), and homogeneity of regression slopes (*p* = .0990) were met. After adjusting for horse bodyweight, there was a difference between the groups (*p* = .0008, partial *η* ^2^ = .53). Horse weight was not associated with maximum strength (*p* = .4007, partial *η* ^2^ = .03). The control group demonstrated a higher adjusted mean maximum strength of 11.39 (±0.72) kN compared with CS with an adjusted mean of 6.70 (±0.72) kN, with a mean difference of 4.69 kN. Similarly, the control group exhibited higher maximum strength than XP with an adjusted mean of 8.00 (±0.75) kN, with a mean difference of 3.39 kN.

**FIGURE 8:**
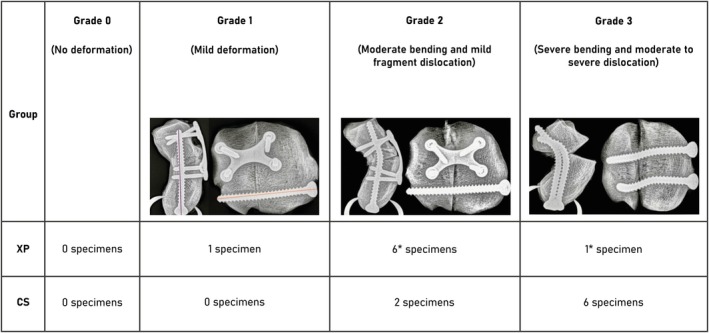
Summary of failure mode grades for specimens in the cortical screw (CS) and X‐plate (XP) groups. With additional representation of the degree of screw bending following biomechanical testing, shown in proximodistal and lateromedial radiographic views. For three XP specimens, one evaluator classified bending as Grade 3, whereas the remaining evaluators rated it as Grade 2. *One XP specimen was rated as Grade 2 by two evaluators and as Grade 3 by the other two evaluators. For one CS specimen, one evaluator assigned Grade 2, while the remaining evaluators classified it as Grade 3.

Almost all implants exhibited plastic deformation, although no breakage or pull‐out of the screws or plates was evident. One XP‐specimen showed no plastic deformation and was subsequently excluded from the statistical comparison. According to Landis et al. the strength of agreement among the evaluators was “substantial” (Fleiss' kappa is 0.736).^22^ This high level of interrater agreement supported further statistical comparison between Group CS and Group XP. All specimens in Group CS were categorized as Grade 2 or 3, whereas specimens in Group XP were predominantly classified as Grade 2 (Figure 8). Failure mode was more often classified as grade 3 in the specimens repaired with two cortical screws compared to those repaired with one cortical screw and an X‐plate (*p* = 1.074e‐7) with a strong effect size of *d* = 0.677.^29^ The X‐plate itself showed no visible deformation.

The mean fracture gap size was 4.1 (±3.71) mm. Seven specimens were less than 3 mm. Six specimens measured between 3 and 6 mm and three specimens more than 6 mm. Specimens repaired with two cortical screws (Group CS) showed a larger fracture gap after force to failure testing compared to those repaired with one cortical screw and X‐plate (Group XP) (*p* = .0039) with a strong effect size of *d* = 1.724.^29^

## 4 DISCUSSION

This study is the first to biomechanically evaluate two different fixation methods for vertical plane fractures of the accessory carpal bone, comparing fixation with two 4.5 mm cortical screws in lag fashion to a construct combining one 4.5 mm cortical screw in lag fashion and a medium angular stable X‐plate with four 2.7 mm locking screws.

Considering all aspects of surgical technique, including surgical approach, larger extent of soft tissue dissection, fracture reduction, intraoperative imaging, margin for error, and overall technical difficulty, the authors consider the X‐plate technique to offer practical advantages. Placement of two cortical screws in lag fashion remains technically demanding and carries a risk of breaching the medial or lateral cortex, potentially leading to soft tissue damage or reduced construct stability. Although screw insertion is guided by imaging modalities, accurately estimating the exact direction of the drill can be difficult, as it depends on the projection angle. In contrast, application of a laterally positioned X‐plate allows plate positioning under direct visual guidance, supplemented by imaging, and provides additional axial stability. Although the study hypothesis that an X‐plate combined with a single cortical screw would provide greater mechanical stability than two cortical screws was not supported, the X‐plate configuration demonstrated equivalent biomechanical performance while being technically less demanding. These findings suggest that, in clinical practice, the X‐plate technique may represent a feasible alternative when fixation with two lag screws is challenging or carries a higher risk of complications.

Both fixation constructs demonstrated comparable stiffness and maximum strength, even when accounting for the bodyweight of the horses. Constructs incorporating the X‐plate, however, tended to show greater stability and lower variability in maximum strength, indicating higher mechanical consistency. This may be attributed to the neutralization of the forces on the tension side of the bone, combined with interfragmentary compression provided by the cortical screw placed in lag fashion. As expected, the maximum strength and stiffness of the repaired bones was lower than that of the native bone, which served as a reference and control rather than a direct comparator for fixation strength.

One specimen in Group XP showed no evidence of implant bending. The lack of plastic deformation was likely due to slippage of the specimen in the fixture near the end of the compression test, when the sample was no longer visible inside the testing machine, resulting in incomplete force transmission to the construct.

The specimen which showed mild bending of the implants after biomechanical testing was repaired using an X‐plate and a cortical screw in lag fashion. In contrast, the authors agreed that the majority of the specimens in Group CS (5 of 8) demonstrated severe bending of the cortical screws. Failure mode analysis revealed a difference in the grade of implant bending between the surgical techniques, as confirmed by a Mann–Whitney U test, with a strong effect size according to Cohen (*r* > 0.5). When considered alongside the compression test results, these findings indicate greater mechanical stability in specimens repaired with the X‐plate and a cortical screw in lag fashion. Macroscopic bone damage was observed in all specimens following biomechanical testing.

The difference in procedure times was primarily attributable to the surgical approach required for each fixation technique. Placement of the X‐plate necessitated more extensive soft tissue dissection, making the procedure more time‐consuming. In contrast, cortical screws were inserted through a stab incision, allowing for a shorter operative time. Time measurement was based on intraoperative radiographs and excluded the skin incision and tendon mobilization, which, according to the authors' experience, required less than 5 min. One outlier in the CS group occurred due to repeated slippage of the reduction forceps, which prolonged the procedure.

The distal position of the cortical screw in Group XP was selected because the bone is thicker and less convex in the distal half of the ACB, facilitating sagittal insertion of the screw. Regardless of the screw position, it was unlikely that the plate screws would interfere with the cortical screw as the locking head screws of the X‐plate are angled towards the center of the plate. The angular stability of the locking screws in the X‐plate allows stable fixation of the fragments even with short screws. This is particularly important because the narrow width of the ACB limits screw length to avoid impingement of the deep flexor tendon in the carpal sheath.

From the 20 fixated ACBs, two samples of each group were excluded from biomechanical testing because of inadequate fixation of the fracture. There is only limited space to place the screw sagittal through the ACB, whereby the individual curvature of the bone must be considered. Screw placement was an additional challenge as it was difficult to consistently repeat the correct tangential projection (D80°Pr30°L‐PaDiMO) of the ACB. Studies on computer‐assisted orthopedic surgery (CAOS) in equine patients have demonstrated high precision, which is particularly valuable for achieving accurate multiplanar intraoperative orientation in orthopedic procedures.^15^, ^30^, ^31^ Given the complex convex shape of the accessory carpal bone, such precision could facilitate optimal screw placement and reduce the risk of cortical breach during fracture repair. As CAOS, which utilizes imaging data and recognition‐guided software, was not available in this study, intraoperative assessment of screw placement relied solely on radiography. In contrast, the position of the X‐plate could be directly visualized during application, allowing more controlled placement.

It is not known whether the simulated forces in our experiment accurately reflect those acting on the ACB in vivo. Case reports indicate that many horses presenting with ACB fractures had sustained a fall.^15^, ^16^, ^32^ Additionally, trauma is reported to occur when the limb is in flexion, causing the ACB to be crushed between the third metacarpal bone and the radius,^3^, ^8^, ^10^, ^33^ a mechanism previously referred to as the “nutcracker” effect.^7^ Other authors hypothesize that traction from ligamentous attachment may also contribute to ACB fractures.^8^, ^11^ After consultation with a biomechanical expert (XX, personal communication), the authors concluded that the greatest stress on the ACB likely occurs when the carpus is in hyperextension.^34^ At maximum carpal loading, the biomechanical forces of the multiple strong ligaments and muscle insertions attached to the ACB are counteracted by the strong retinaculum, resulting in dorsopalmar compressive forces.^34^ Since tensile forces are difficult to replicate experimentally, we have opted for a pure compression model in a palmarodorsal direction for our testing. This approach is supported by a previous study in which native specimens were loaded to failure, producing fractures comparable to those observed in spontaneous cases.^35^, ^36^ In contrast, the study by Launois et al., force‐to‐failure compression tests were performed laterally to medially using a double‐column electromechanical press.^20^

A technical limitation of the biomechanical testing was the need to precondition the specimens, which may have resulted in slight damage to the bone prior to the actual compression test. This may have affected the overall maximum strength. In addition, washers were used on the palmar and dorsal sides to stabilize the specimen in the testing machine and to distribute the pressure over the bone surface rather than the screw head. However, it cannot be ruled out that force was transmitted directly to the cortical screws. This could explain the higher dispersion of values in the lag screw group.

In conclusion, although neither fixation method proved biomechanically superior in compression testing, the use of an X‐plate combined with a cortical screw in lag fashion offered greater technical advantages and ease of application while providing comparable stability. These findings suggest that repairing a vertical plane fracture of the ACB with a single lag screw and an X‐plate may be a justified alternative to two lag screws. Although the X‐plate technique required a more extensive soft tissue dissection than the insertion of two cortical screws through stab incisions, this study cannot determine whether the larger surgical approach would increase the risk of postoperative complications. As both techniques involve implant placement adjacent to critical soft tissue structures, the potential for postoperative morbidity is likely comparable. However, the extent of tissue handling and exposure required for X‐plate application may theoretically increase the risk of local inflammation or surgical site infection. In cases of comminuted fractures of the ACB, where cortical screws in lag fashion are not feasible, the authors acknowledge that the application of an X‐plate may also have limitations with respect to effectively bridging multiple fracture lines. Further research is warranted to investigate whether alternative implant designs, such as plates with larger screw diameters or customized implants tailored to the anatomy of the ACB, could enhance construct stability and improve surgical outcomes.

## AUTHOR CONTRIBUTIONS

Gernhardt J, DVM: Contributed to the design of the study, assisted surgical management and biomechanical testing, diagnostic imaging, compiled all data, drafted, and revised the manuscript. Reuter T, DING, ME: Contributed to the design of the study, performed biomechanical testing, analyzed, and interpreted data for statistical significance. Mählmann K, DVM, DECVS: Review and editing by overseeing data collection, interpreting the data, and performing scientific in‐line editing of the manuscript. Schulze N, DVM, PhD: Contributed to the design of the study, assisted surgical management of the cases, diagnostic imaging and in‐line editing of the manuscript. Lischer CJ, DVM, DECVS: Contributed to the design of the study, was responsible for the surgical management, review and editing by overseeing data collection, diagnostic imaging, interpreted data, and provided scientific in‐line editing of the manuscript. All authors provided a critical review of the manuscript and endorse the final version. All authors are aware of their respective contributions and have confidence in the integrity of all contributions.

## CONFLICT OF INTEREST STATEMENT

The authors declare no conflicts of interest related to this report.

## Supporting information


**Figure S1.** Summary of quasi‐static uniaxial compression tests in palmarodorsal direction of the accessory carpal bone (ACB) to determine the maximum strength (force to failure) with integrated gradient determination (blue line) for the native bone (Control Group CG).

**Figure S2.** Summary of quasi‐static uniaxial compression tests in palmarodorsal direction of the accessory carpal bone (ACB) to determine the maximum strength (force to failure) with integrated gradient determination (blue line) for the fracture fixation with cortical screws in lag fashion (CS).

**Figure S3.** Summary of quasi‐static uniaxial compression tests in palmarodorsal direction of the accessory carpal bone (ACB) to determine the maximum strength (force to failure) with integrated gradient determination (blue line) for the fracture fixation with X‐plate and cortical screw in lag fashion (XP).
