# Supplementary material for: Comparative biomechanical analysis of equine accessory carpal bone fracture repair: Cortical screws in lag fashion versus X‐plate technique
Source: Vet Surg. 2025 Dec 21;55(3):620–30. doi: 10.1111/vsu.70071 (PMC13069204; doi:10.1111/vsu.70071)
Supplement: Supplementary file 1 — Figure S1. Summary of quasi‐static uniaxial compression tests in palmarodorsal direction of the accessory carpal bone (ACB) to determine the maximum strength (force to failure) with integrated gradient determination (blue line) for the native bone (Control Group CG). [file VSU-55-620-s003.docx]

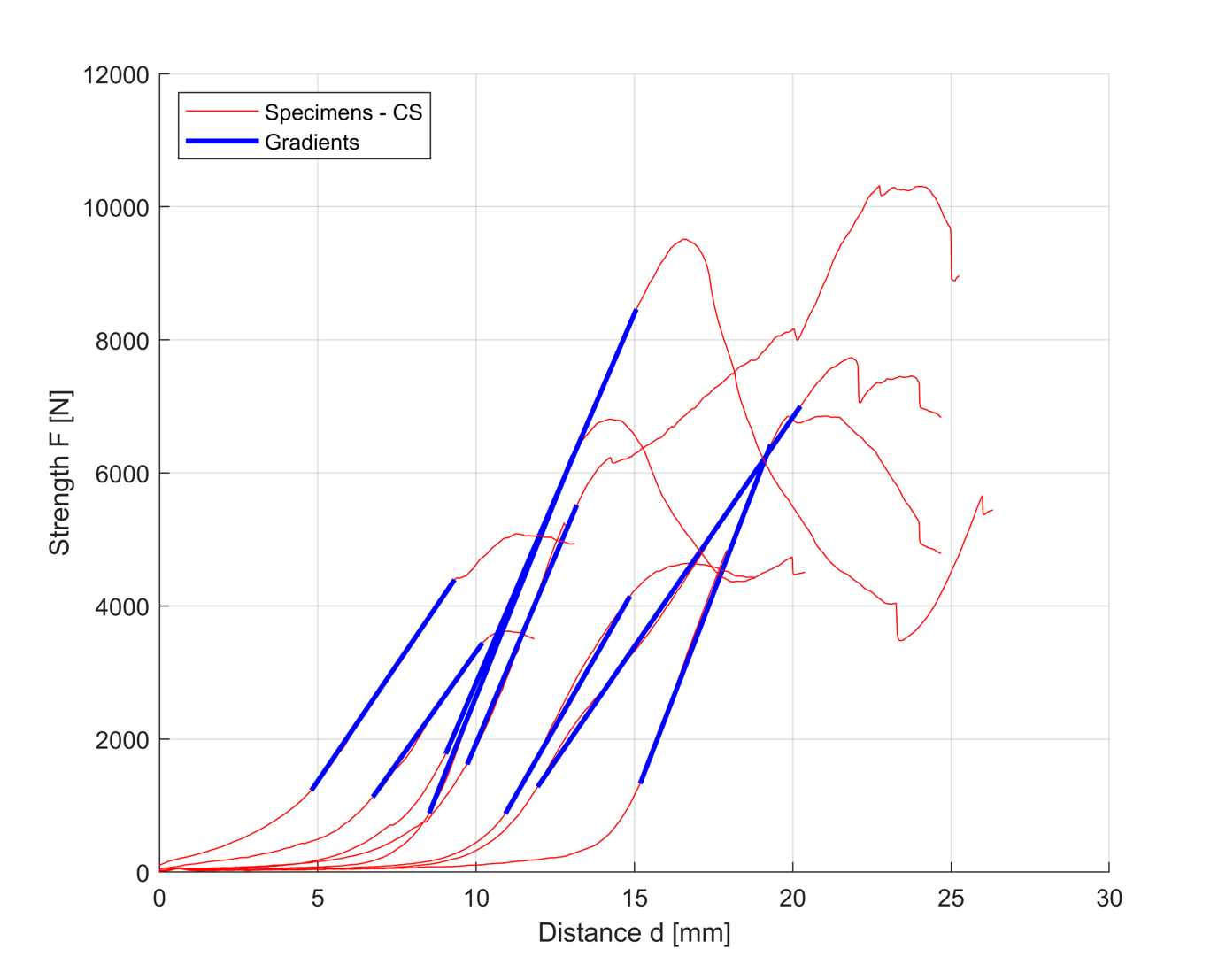

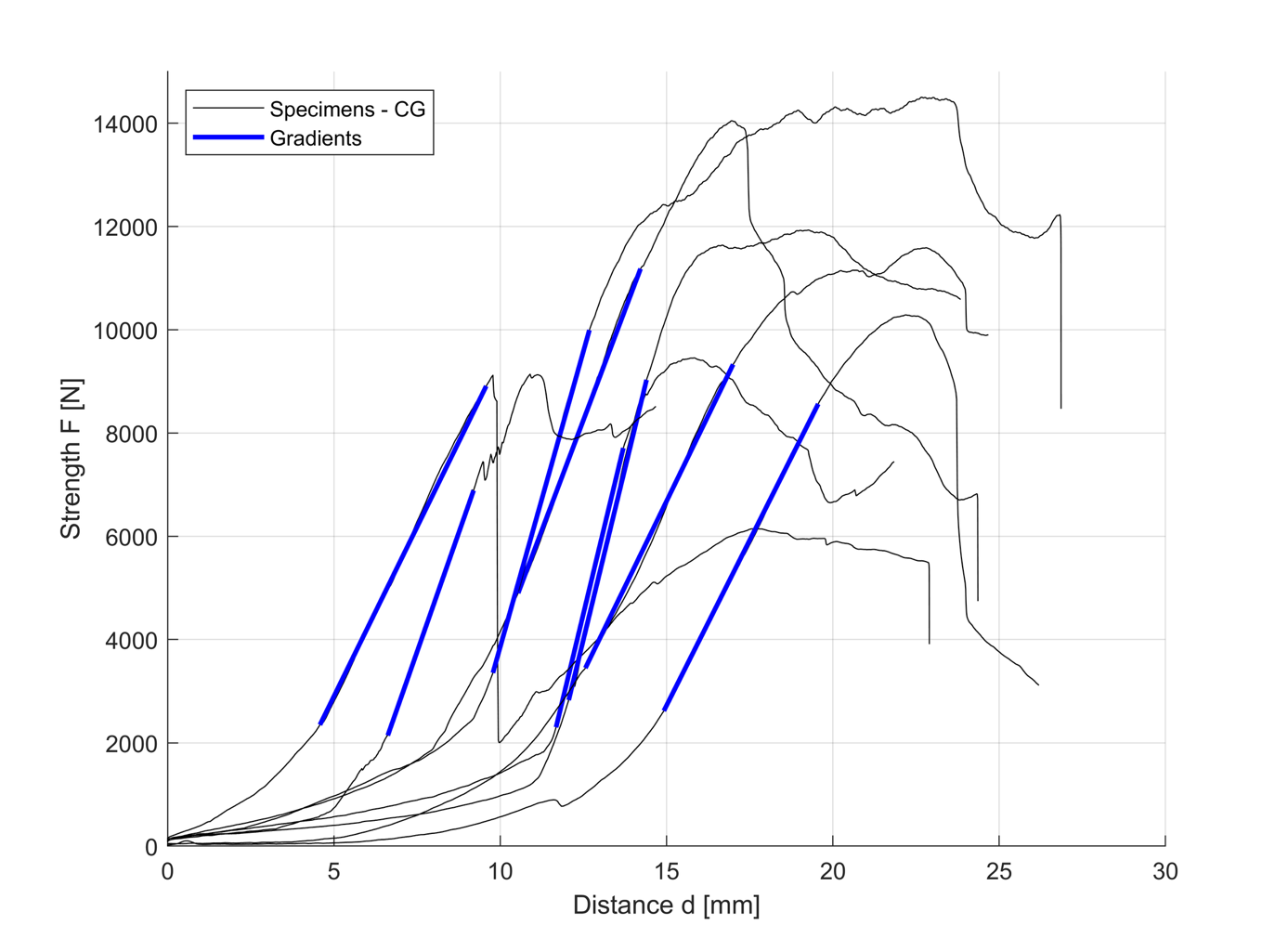


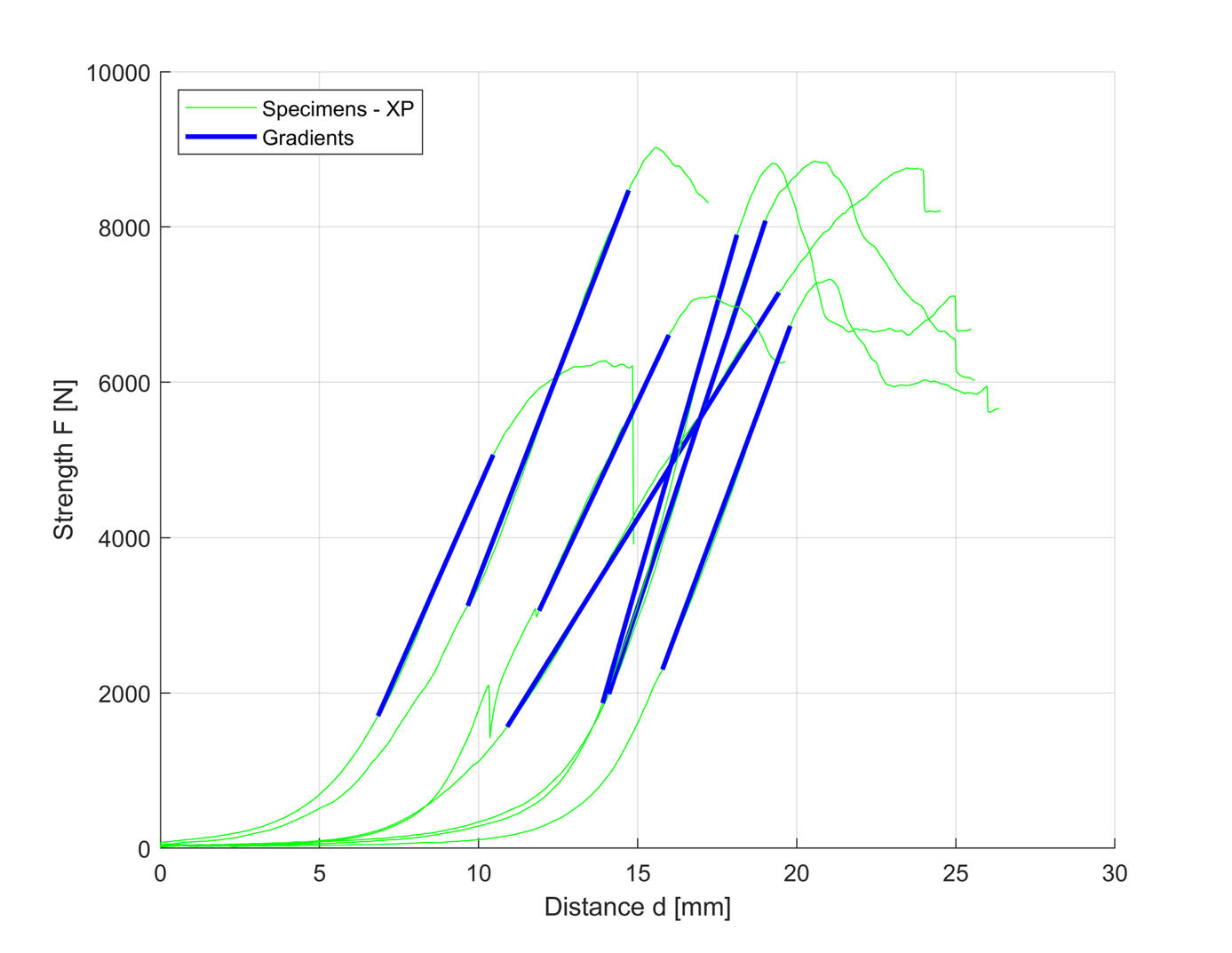


Supplement 1:

Summary of quasi-static uniaxial compression tests in palmarodorsal direction of the ACB to determine the maximum strength (force to failure) with integrated gradient determination (blue line) for the native bone (Control Group CG).

Supplement 2:

Summary of quasi-static uniaxial compression tests in palmarodorsal direction of the ACB to determine the maximum strength (force to failure) with integrated gradient determination (blue line) for the fracture fixation with cortical screws in lag fashion (CS).

Supplement 3:

Summary of quasi-static uniaxial compression tests in palmarodorsal direction of the ACB to determine the maximum strength (force to failure) with integrated gradient determination (blue line) for the fracture fixation with X-plate and cortical screw in lag fashion (XP).
