# Supplementary material for: Comparative biomechanical analysis of equine accessory carpal bone fracture repair: Cortical screws in lag fashion versus X‐plate technique
Source: Vet Surg. 2025 Dec 21;55(3):620–30. doi: 10.1111/vsu.70071 (PMC13069204; doi:10.1111/vsu.70071)
Supplement: Supplementary file 2 — Figure S2. Summary of quasi‐static uniaxial compression tests in palmarodorsal direction of the accessory carpal bone (ACB) to determine the maximum strength (force to failure) with integrated gradient determination (blue line) for the fracture fixation with cortical screws in lag fashion (CS). [file VSU-55-620-s001.docx]

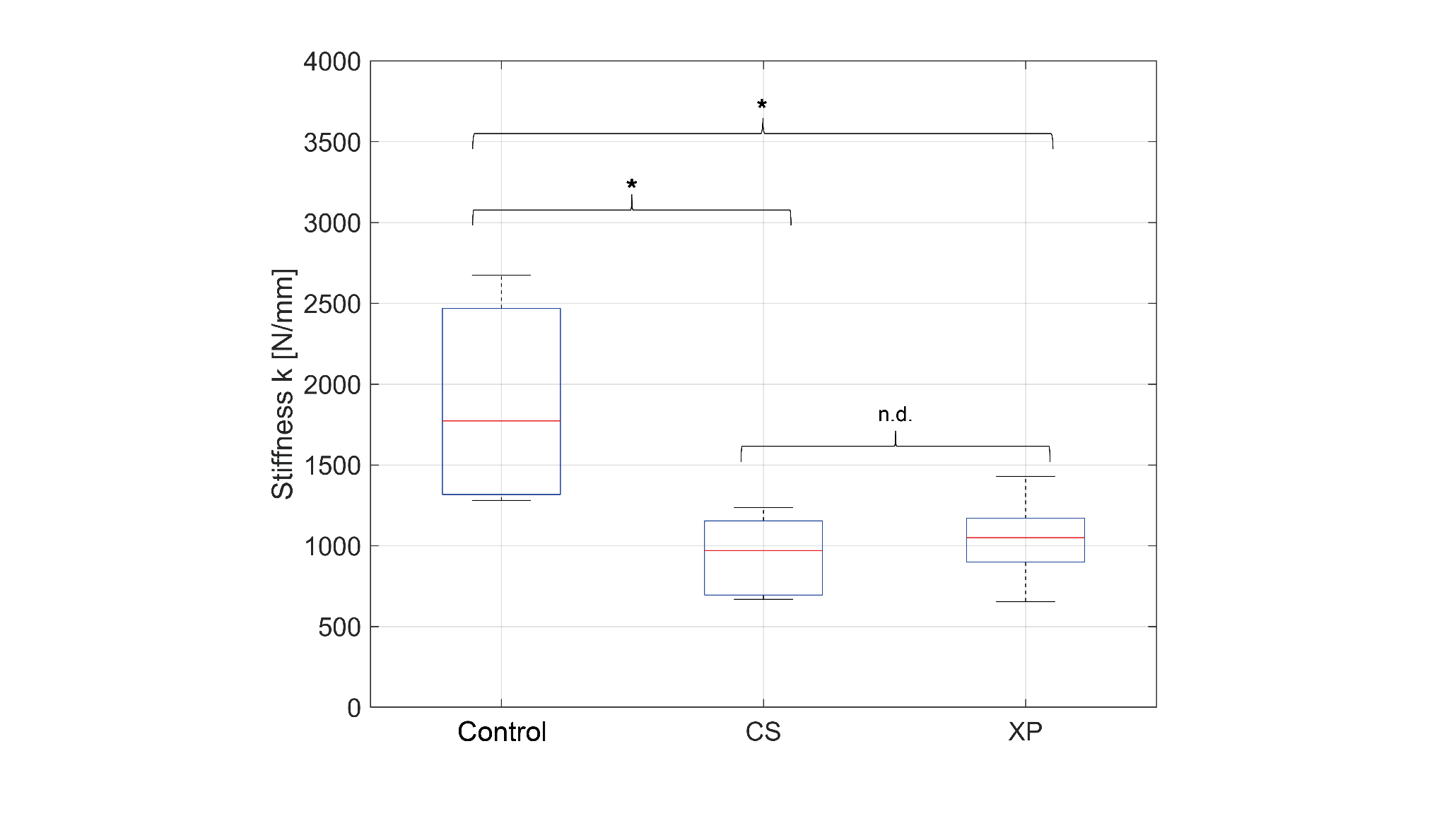


Supplement 2: Comparison of the statistical distribution of maximum stiffness (force to failure) between control group, fracture fixation with cortical screws in lag fashion (CS) and X-plate and cortical screw in lag fashion (XP). (n.d. - no difference P > 0.05; * - significant differences P < 0.05; P value adjustment method: Bonferroni).
