# Supplementary material for: Comparative biomechanical analysis of equine accessory carpal bone fracture repair: Cortical screws in lag fashion versus X‐plate technique
Source: Vet Surg. 2025 Dec 21;55(3):620–30. doi: 10.1111/vsu.70071 (PMC13069204; doi:10.1111/vsu.70071)
Supplement: Supplementary file 3 — Figure S3. Summary of quasi‐static uniaxial compression tests in palmarodorsal direction of the accessory carpal bone (ACB) to determine the maximum strength (force to failure) with integrated gradient determination (blue line) for the fracture fixation with X‐plate and cortical screw in lag fashion (XP). [file VSU-55-620-s002.docx]

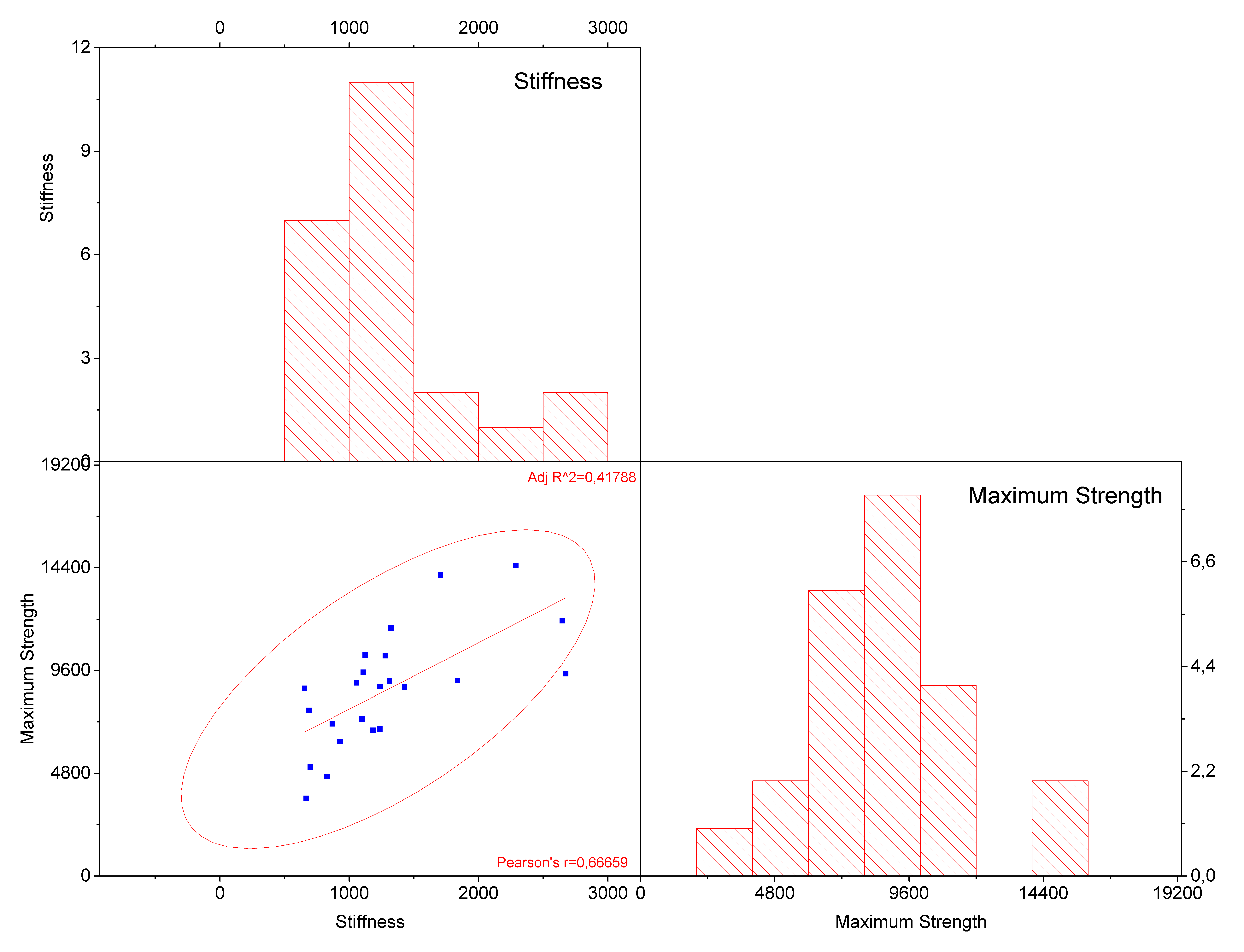


Suppement 3: Statistical evaluation of the relationship between stiffness and maximum strength using linear correlation (red line, r = 0.667) and distribution analysis (red circle and histogram).
